# Trajectories of Physical and Pharmacological Dependence in Previously Independent Nonagenarian Sepsis Survivors

**DOI:** 10.1111/ggi.70545

**Published:** 2026-05-17

**Authors:** Yuichiro Shimoyama, Noriko Kadono, Osamu Umegaki

**Affiliations:** ^1^ Department of Anesthesiology, Osaka Medical and Pharmaceutical University, Intensive Care Unit Osaka Medical and Pharmaceutical University Hospital Takatsuki Osaka Japan

1

As advancements in critical care have improved short‐term survival among the oldest‐old, establishing true “success” requires evaluating the long‐term trajectory of Post‐Intensive Care Syndrome (PICS) [1, 2, 3]. Mortality is well‐documented, but the comprehensive loss of baseline autonomy in extreme old age remains poorly quantified. We investigated the 6‐month post‐ICU trajectories of physical and pharmacological dependence in a cohort of previously independent nonagenarian sepsis survivors. We specifically focused on severe sepsis because it is a primary driver of ICU admissions and represents the ultimate physiological stress test, unleashing profound systemic inflammation that damages skeletal muscle and the central nervous system, thereby acting as a catastrophic accelerator of senescence.

Using the National Database of Japan (NDB), which captures > 98% of national health insurance claims [4], we conducted a retrospective cohort study of nonagenarian (aged ≥ 90 years) sepsis survivors admitted to intensive care units between 2015 and 2020. To isolate the impact of critical illness from baseline senescence, all 269 included patients were strictly phenotype‐matched: living completely independently, with zero claims for home medical care, rehabilitation, or central nervous system (CNS)‐active medications for at least 6 months prior to ICU admission.

We evaluated the 6‐month post‐ICU incidence of physical decline (new initiation of home medical care) and new pharmacological dependence. Among the 269 strictly independent survivors (median age category, 90–94 years; 91 [33.8%] required mechanical ventilation and < 10 required renal replacement therapy), the loss of baseline autonomy was substantial (Figure 1). A total of 85 (31.6%) patients suffered physical decline requiring new home medical care. Concurrently, 83 (30.9%) patients developed profound distress necessitating new prescriptions of CNS‐active medications. The breakdown of these CNS prescriptions (including frequent overlapping prescriptions) was as follows: antipsychotics (*n* = 59), antidepressants (*n* = 59), hypnotics (*n* = 16), and anti‐dementia drugs (*n* = 11). Crucially, 34 (12.6%) patients experienced a concurrent decline in both domains.

**FIGURE 1:**
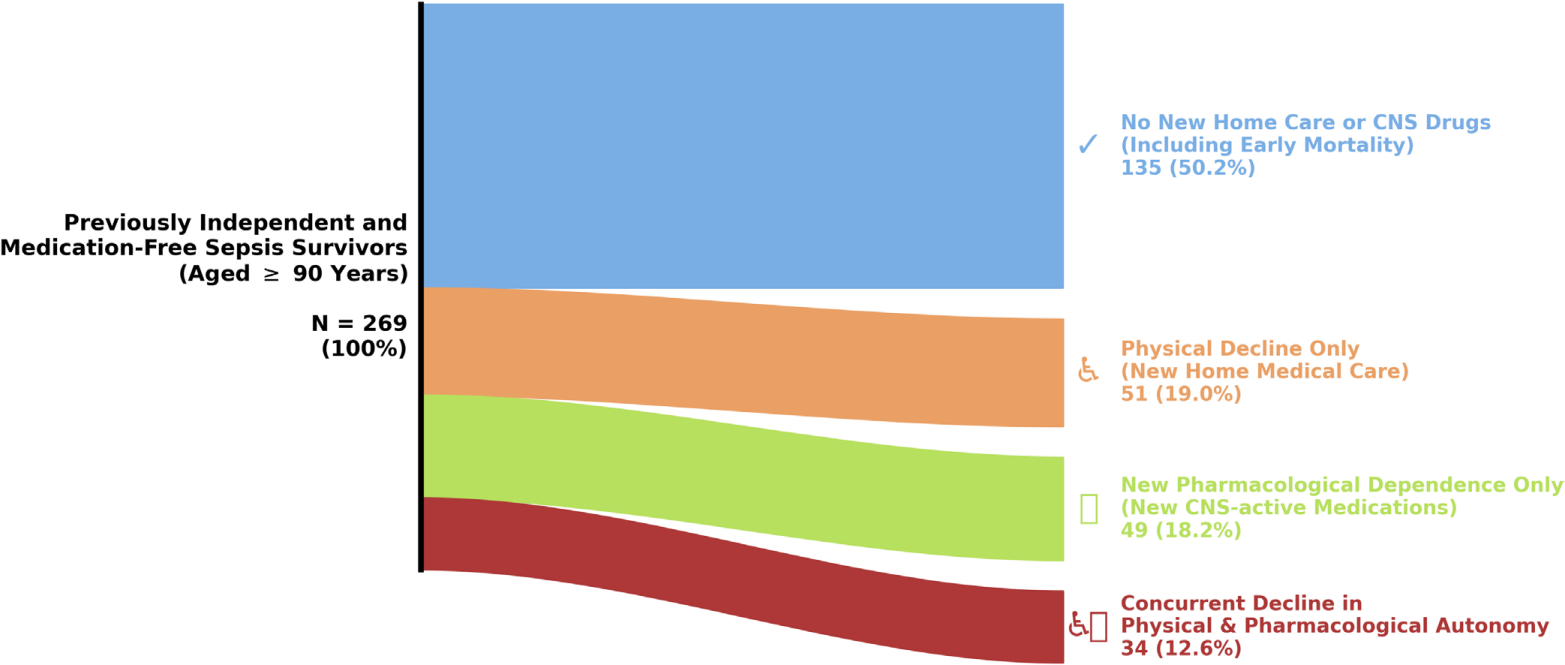
6‐Month trajectories post‐ICU: Sankey diagram illustrating the 6‐month post‐ICU trajectories of 269 previously independent and medication‐free nonagenarian sepsis survivors. Intuitive pictograms are included next to each trajectory to facilitate immediate visual comprehension. Despite surviving the acute critical illness, nearly half of the cohort lost their baseline autonomy, transitioning into physical decline, new pharmacological dependence (new initiation of CNS‐active medications including antipsychotics, antidepressants, anti‐dementia drugs, and hypnotics), or a concurrent decline in both domains. Only 50.2% avoided new home medical care or CNS drugs, an optimistic estimate that inherently includes early out‐of‐hospital mortality.

Ultimately, only 135 (50.2%) patients were categorized as requiring “No New Home Care or CNS Drugs.” However, because administrative claims cannot track early out‐of‐hospital mortality, this group inherently includes patients who died before generating new claims. Given that a recent nationwide study in Japan reported an in‐hospital mortality rate of 24.9% for sepsis patients aged ≥ 90 years [5], the true rate of intact survival is definitively and substantially lower than 50.2%.

Returning to a completely independent life represents a nearly 50% probability at best for previously robust nonagenarians. While nonagenarians inherently exhibit a high baseline rate of natural functional decline, the abrupt loss of autonomy observed within a mere 6‐month window post‐ICU underscores the substantial intervention burden of critical illness [6]. Critical care for patients aged ≥ 90 years must pivot toward a holistic approach prioritizing the preservation of autonomy. Beyond life‐saving measures, implementing early mobilization and strictly adhering to the ABCDEF bundle to prevent over‐sedation are imperative to mitigate subsequent functional and cognitive exhaustion. Furthermore, tracking the trajectory of intermediate phenotypes during the acute phase—such as biomarkers of myocardial injury, acute kidney injury, and profound systemic inflammation—may serve as crucial early predictors for long‐term loss of autonomy.

## Author Contributions

**Yuichiro Shimoyama:** conceptualization, methodology, formal analysis, investigation, writing – original draft, visualization. **Noriko Kadono:** methodology, validation, writing – review and editing. **Osamu Umegaki:** supervision, validation, writing – review and editing.

## Funding

This work was supported by Japan Society for the Promotion of Science (JP21K09088).

## Ethics Statement

The study protocol was approved by the Ethics Committee of Osaka Medical and Pharmaceutical University (Approval No. 2871‐2, approved on February 19, 2020) and the Ministry of Health, Labour and Welfare of Japan. The study was performed in accordance with the ethical standards as laid down in the 1964 Declaration of Helsinki and its later amendments. Given the retrospective nature of the study and the use of anonymized administrative data, the requirement for informed consent was waived by the Ethics Committee.

## Conflicts of Interest

The authors declare no conflicts of interest.

## Data Availability

The data supporting this study are not publicly available due to strict privacy restrictions under Japanese law. While the authors cannot share the data, eligible researchers may apply directly to the Japanese Ministry of Health, Labour and Welfare (MHLW) for access.
